# Evolution of Porcine Kobuvirus Infection, Hungary

**DOI:** 10.3201/eid1604.090937

**Published:** 2010-04

**Authors:** Gábor Reuter, Sándor Kecskeméti, Péter Pankovics

**Affiliations:** ÁNTSZ Regional Institute of State Public Health Service, Pécs, Hungary (G. Reuter, P. Pankovics); Central Agricultural Office, Debrecen, Hungary (S. Kecskeméti)

**Keywords:** Kobuvirus, endemic infection, viremia, interhost evolution, mutation, domestic pig, viruses, Hungary, dispatch

## Abstract

Porcine kobuvirus was first identified in early 2007 in Hungary. Originally thought to be confined to the intestine, almost 2 years later the virus was found in the blood of clinically healthy pigs on the same farm. Porcine kobuvirus may be widely distributed on pig farms worldwide.

Picornaviruses (family *Picornaviridae*) are small, nonenveloped viruses with single-stranded, positive-sense genomic RNA; they are divided into 12 genera: *Aphthovirus, Avihepatovirus, Cardiovirus, Enterovirus, Erbovirus, Hepatovirus, Parechovirus, Sapelovirus, Senecavirus, Teschovirus, Tremovirus*, and *Kobuvirus* (*1*). The genus *Kobuvirus* consists of 2 officially recognized species, *Aichi virus* and *Bovine kobuvirus,* and 1 candidate species, porcine kobuvirus (*2*–*4*). Aichi virus (strain A846/88) was first isolated in 1989 in Japan, from a fecal sample from a person with acute gastroenteritis (*2*). Aichi viruses have also been detected in human fecal samples in other countries in Asia (*5*); Europe (*6*,*7*), including Hungary; South America (*6*); and North Africa (*8*). Bovine kobuvirus (strain U-1) has been detected in culture medium derived from cattle serum suspected to be contaminated with cattle feces (*3*) and in fecal samples from cattle in 2003 in Japan (*3*) and in 2008 in Thailand (*9*) and Hungary (*10*).

Porcine kobuvirus was first identified and the complete genome (S-1-HUN; EU787450) characterized from fecal samples from domestic pigs in Hungary (*4*,*11*). Porcine kobuvirus has also been recently (2009) reported from the People’s Republic of China (*12*). Kobuvirus genomes are ≈8.2–8.4 kb. Genome organization includes leader (L) protein following structural (VP0, VP3, and VP1) and nonstructural (2A-2C and 3A-3D) regions (*1*,*3*,*4*,*11*). Genetic identity on the coding region between Aichi, U-1, and S-1-HUN viruses varies from 35% (L protein) to 74% (3D region) (*3*,*11*).

Kobuvirus infection has been thought to be confined to the intestine. To our knowledge, detection of kobuvirus in the infected host species serum (viremia) has not been reported. We report the endemic circulation and in vivo evolution of porcine kobuvirus on a pig farm where the virus was originally discovered and virus escape from the gastrointestinal tract, resulting in viremia in domestic pigs.

## The Study

In February 2007, a total of 39 (65%) of 60 fecal samples from clinically healthy domestic pigs on a farm in eastern Hungary were positive (by reverse transcription–PCR) for porcine kobuvirus (*11*) (Table 1). In November 2008, 21 months later, we obtained 60 fecal and 60 serum sample pairs, again from apparently clinically healthy domestic pigs on the same farm. We divided these pigs, all <6 months of age with no diarrhea, into 4 age groups (Table 1). Blood samples were taken from the jugular vein by using closed vacuum sets. Fecal and serum samples were processed and tested separately for porcine kobuvirus by using the same methods (RNA isolation, reverse transcription–PCR) and the same primers for screening (UNIV-kobu-F/UNIV-kobu-R for 3D) and for the complete genome analysis described previously (*11*). PCR products were sequenced directly in both directions with the BigDye Reaction Kit (Applied Biosystems, Warrington, UK) by using the PCR primers and an automated sequencer (ABI PRISM 310 Genetic Analyzer; Applied Biosystems, Stafford, TX, USA). The genome was assigned GenBank accession no. GQ249161. Kobuvirus-positive fecal samples were inoculated into Vero cells.

**Table 1:** Incidence of porcine kobuvirus RNA in samples from domestic pigs, Hungary*

Pig age	Specimen, by date			
	2007 Feb		2008 Nov	
	Feces		Feces	Serum
<10 d	15 (100)		8 (53.3)	1 (6.7)
<3–4 wk	14 (93.3)		13 (86.7)	7 (46.7)
<3 mo	3 (20)		5 (33.3)	0
<6 mo	7 (46.7)		6 (40)	8 (53.3)
Total	39/60 (65)		32/60 (53.3)	16/60 (26.6)
*Values are no. (%) or no. positive/no. tested (%). All animals were on the same farm. For each type of sample and age group, 15 specimens were tested. In November 2008, specimens (feces and serum) were collected in pairs from the same animals.				

Porcine kobuvirus RNA was detected in 32 (53.3%) of the 60 fecal samples and 16 (26.6%) of the 60 serum samples collected in November 2008 (Table 1). Porcine kobuvirus was found in at least 1 fecal sample from each age group; from serum samples, viral RNA was detected in 1 sample from the 10-day group, 7 samples from the 3–4-week group, and 8 samples from the 6-month group. Porcine kobuvirus was identified in 9 sample pairs (feces and serum) collected at the same time. Only the serum or fecal samples were kobuvirus positive, for 7 and 23 animals, respectively. The 173-nt sequences of kobuvirus 3D region were genetically identical in all serum and fecal samples collected in 2007 and 2008.

The complete genetic sequence of strain kobuvirus/swine/K-30-HUN/2008/Hungary, detected in a fecal specimen from a 3–4-week-old pig in 2008, was determined and compared with the prototype strain kobuvirus/swine/S-1-HUN/2007/Hungary (Table 2). In the nonstructural 2A-3D regions, 74 (1.7%; 1.14%–2.43% between 3D and 3C) nucleotide substitutions could be detected between the prototype strains collected in 2007 (S-1-HUN) and 2008 (K-30-HUN), which led to 13 (0.9%) nonsynonymous amino acid changes (2, 2, 5, 2, and 2 aa in the 2A, 2B, 2C, 3C, and 3D regions). In structural regions, 36 (1.42%) nt substitutions were detected, leading to 6 (0.71%) nonsynonymous amino acid changes (3–3 aa in VP0 and VP3 regions). The highest and the lowest rates of nucleotide mutations were seen in coding regions, L and 3D, respectively (Table 2). For amino acid levels, the highest changes in percentage within the 21-month period were found in 2C (1.49%), 2A (1.47%), and VP3 (1.34%) (Table 2). No amino acid changes were detected in regions VP1, 3A, and 3B. No cytopathic effect was seen in Vero cells after serial passages.

**Table 2:** Natural interhost in vivo mutation changes in sequences of 2 porcine kobuvirus strains, Hungary*

Region	Nucleotides				Amino acids		
	Length	Difference	% Difference		Length	Difference	% Difference
5′ UTR	576	1	0.17		–	–	–
L	585	15	2.56		195	1	0.51
VP0	1,098	19	1.73		366	3	0.82
VP3	669	8	1.19		223	3	1.34
VP1	762	9	1.18		254	0	0
2A	408	6	1.47		136	2	1.47
2B	585	7	1.19		195	2	1.02
2C	1,005	23	2.28		335	5	1.49
3A	270	6	2.22		90	0	0
3B	102	2	1.96		34	0	0
3C	576	14	2.43		192	2	1.04
3D	1,407	16	1.14		469	2	0.42
3′ UTR	167	1	0.6		–	–	–
Nonstructural†	4,353	74	1.7		1,451	13	0.89
Structural‡	2,529	36	1.42		843	6	0.71
Complete genome	8,210	127	1.54		2,489	20	0.80

*Strain length shown is for kobuvirus/swine/K-30-HUN/2008/Hungary (accession no. GQ249161) and is compared with prototype strain kobuvirus/swine/S-1-HUN/2007/Hungary (length not shown; accession no. EU787450), from samples collected from pigs on the same farm, November 2008 and February 2007 respectively. UTR, untranslated region; L, leader region. †2A-3D. ‡VP0–VP1.

## Conclusions

We report the endemic circulation and natural interhost evolution of porcine kobuvirus among domestic pigs on a pig farm within a 21-month period. This follow-up study indicates that domestic pigs are generally infected with porcine kobuvirus. It is likely that porcine kobuvirus is not restricted geographically and is widely distributed on pig farms worldwide. According to a veterinarian’s clinical report, no clinical signs were associated with the endemic kobuvirus infection. In this closed-animal community, the susceptible young host generations were continuously infected; the length of the cycle interval when the virus is outside the host (nonreplicative viral form) is probably short. However, the number of nucleotide changes is consistent with rates observed for other picornaviruses (*13*–*15*), suggesting good adaptation of the virus–host relationship. This adaptation is also supported by the level of the nucleotide changes in different gene regions. More nucleotide mutations were found in nonstructural than in structural regions, which leads to a nearly equal probability for nonsynonymous amino acid changes in these 2 regions.

No available data confirm transmission mode, transmission efficiency, and pathogenesis of kobuviruses. Aichi virus has been reported to be shed in feces and maybe transmitted by food (especially seafood) in humans (*2*,*5*,*7*), suggesting fecal–oral transmission. However, in the pigs, viral RNA or infectious kobuvirus particles were also present in serum of virus-infected pigs. This finding suggests that porcine kobuvirus (and possibly kobuviruses in general) escaped the gastrointestinal tract into the circulatory system in immunocompetent virus-infected hosts, resulting in viremia. Further study is needed to investigate whether the viremia was acquired passively or actively. In addition to direct fecal–oral transmission, the possibility of kobuvirus transmission through breast-feeding (milk) and of blood-borne, foodborne, and zoonotic infections remains.

Similar explanations for the pathogenesis of many picornaviruses exist. Escape from the gastrointestinal tract into the bloodstream was probably the situation for the bovine kobuvirus detected in culture medium supplemented with cattle serum (*3*), which was suspected of possibly being contaminated with feces containing bovine kobuviruses. Porcine kobuvirus viremia open these data to another interpretation for bovine kobuvirus infections and for Aichi virus pathogenesis in humans. Aichi virus was identified in low incidence in fecal samples from humans with gastroenteritis, but seroprevalence was high (*6*,*7*). Knowledge about viremia and natural virus evolution is crucial for understanding the pathogenesis, transmission, immunology, and clinical manifestations of kobuvirus infection in general and especially in humans.
